# Health system delay in pulmonary tuberculosis treatment in a country with an intermediate burden of tuberculosis: a cross-sectional study

**DOI:** 10.1186/1471-2458-13-250

**Published:** 2013-03-21

**Authors:** Anamarija Jurcev-Savicevic, Rosanda Mulic, Karlo Kozul, Bozica Ban, Jasna Valic, Ljiljana Bacun-Ivcek, Ivan Gudelj, Gordana Popijac-Cesar, Snjezana Marinovic-Dunatov, Aleksandar Simunovic

**Affiliations:** 1 Teaching Public Health Institute of Split and Dalmatia County and School of Medicine, University of Split, Vukovarska 46, 21 000 Split, Croatia; 2 School of Medicine, University of Split, Soltanska 2, 21000 Split, Croatia; 3 Public Health Institute of Osječko-Baranjska County, F. Krežme 1, 31000 Osijek, Croatia; 4 Public Health Institute “Dr Andrija Štampar”, Mirogojska cesta 16, 10000 Zagreb, Croatia; 5 Public Health Institute of Istarska County, Nazorova 23, 52100 Pula, Croatia; 6 Department of pulmonary diseases, Split University Hospital, Spinčićeva 1, 21000 Split, Croatia; 7 Public Health Institute of Krapinsko-Zagorska County, Ivana Gorana Kovačića 1, 49250 Zlatar, Croatia; 8 Public Health Institute of Zadarska County, Kolovare 2, 23000, Zadar, Croatia; 9 Croatian National Institute of Public Health, Rockefellerova 7, 10000 Zagreb, Croatia

**Keywords:** Tuberculosis, Croatia, Delay, Health system

## Abstract

**Background:**

Delayed diagnosis and treatment of tuberculosis increase both the severity of the disease and the duration of infectivity. A number of studies have addressed the issue of health system delays in the treatment of tuberculosis, but mostly in countries with a high or low incidence of the disease. Our understanding of delay is quite limited in settings with an intermediate burden of tuberculosis. We explore the duration and factors associated with delays in the Croatian health system which has free health care and a sufficient network of health services providing tuberculosis diagnosis and care.

**Methods:**

A total of 241 consecutive adults with culture-confirmed pulmonary tuberculosis were interviewed in seven randomly selected Croatian counties and their medical records were evaluated. A health system delay was defined as the number of days from the first consultation with a physician to the initiation of anti-tuberculosis treatment. A long delay was defined as a period exceeding the median delay, while an extreme delay was considered to be above the 75^th^ percentile delay.

**Results:**

The median health system delay was 15 days while the 75^th^ percentile was 42 days (the 5^th^ and 95^th^ percentile being 1 and 105 days respectively). Almost 30% of tuberculosis patients remained undiagnosed for more than 30 days after the initial health care visit. Female patients (p = 0.005), patients with a negative sputum smear (p = 0.002) and patients having symptoms other than the usual ones (0.027) were found to be in significant correlation with a long delay. In a multivariate model, a long delay remained associated with the same variables (p = 0.008, p = 0.003, and p = 0.037, respectively).

A significant association was demonstrated between both the female gender (p = 0.042) and a negative sputum smear (p < 0.001) and extreme delay, while only a negative sputum smear (p < 0.001) remained significant in the multivariate analysis.

**Conclusions:**

Our findings suggest that some groups of tuberculosis patients experienced a health system delay. In such a setting where tuberculosis incidence is decreasing, which leads to a lack of physician experience and expertise, training in tuberculosis is required. Such measure may be useful in reducing the number of missed opportunities for tuberculosis diagnosis.

## Background

Prompt diagnosis and early treatment of tuberculosis (TB) constitute the most effective intervention in controlling and reducing the transmission of *Mycobacterium tuberculosis.* It is generally assumed that a delay in the diagnosis and treatment of TB increases the severity of the disease and the risk of death and leads to newly infected persons who can become reservoirs of new tuberculosis cases at a future point in time [1]. The timely and accurate diagnosis and treatment of TB are therefore essential. TB diagnosis can be delayed when patients postpone seeking care after the onset of symptoms or when more time is required for health providers to diagnose and treat patients. Since the same variable can move in opposite directions, being a risk factor for one delay and a protective factor for another, it is important to consider the two types of delay separately [2,3]. Health system delays show the level of TB knowledge and the efficiency of a country’s National Tuberculosis Control Programme (NTP). Thus, the importance and significance of this particular issue is important for the evaluation and planning of interventions through a particular NTP [1,4], drafted in line with the sixth component of the Stop TB Strategy [5].

A number of studies have addressed the issue of health system delay in the treatment of tuberculosis, though mostly in high- or low-incidence countries [2,3]. This problem, however, is found to be important in both developed and developing countries. Still, there is no consensus on the acceptable duration of delay. Our understanding of the extent and causative factors of health system delay in medium incidence countries is quite limited. In settings where TB is not frequent, it is usually difficult to achieve a high degree of suspicion and to perform immediate diagnostic procedures for tuberculosis. One of these settings is Croatia, with a TB incidence rate of 19 / 100 000 in 2009 and showing a decreasing trend [6,7].

The potential loss of time in the diagnosis and treatment of TB patients, resulting from health system delays, has never been explored in this country.

A study investigating both types of delay was conducted on the same participants. The results of patient delay have already been published [8]. Identifying the duration and the factors related to the health system delay was the objective of this part of the study.

## Methods

### Setting

Croatian TB services are integrated in the mainstream national health system and rely on general practitioners/family medicine physicians guiding TB patients through the entire procedure and course of treatment. The availability and network of health services providing TB care and diagnosis are believed to be sufficient in Croatia [6,9,10], as revealed in a study on patient delay where the long distance to a health facility was among the very few reasons (1.3% of participants) for delay [8]. A developed network of 14 TB laboratories meets international requirements and each laboratory performs microscopy and culture procedures. The laboratory notification system reports smear-positive results within 24 hours and positive culture results immediately upon cultivation [11]. The Croatian health system allows for as many medical visits as required and for as many tests as the GP finds necessary, free of charge to the patient, in the case of many infectious diseases, including tuberculosis. TB treatment is also free of charge, with the institutional procurement of drugs and an uninterrupted supply of first-line drugs [11]. This explains the common reasons for the health system delay being identified elsewhere [12-14]. Causative laboratory delays and distances to the health provider were, however, not explored in the present study.

### Design

This cross-sectional study was performed in seven Croatian counties between April and December 2006. The idea was to include more than 50% of the Croatian population and more than 50% of TB patients registered in the preceding year. This study was started in eight randomly selected counties out of 21, covering 60.8% of the Croatian population and 53.6% of all registered TB patients in the preceding year. In the course of the study, one county was later excluded along with all interviewees because the investigator was moved to another non-participating county. Therefore, seven counties with 48% of all TB patients and 53.9% of the Croatian population were finally covered. A team of epidemiologists who had been working in TB control interviewed the consecutive TB patients in hospitals or in their homes, and reviewed their medical records within one month of the diagnosis of TB. The questionnaire was pre-tested for clarity and consistency, and modified accordingly. Data on demographic, socioeconomic, a high-risk life style and diagnostic factors were collected. Smoking status was classified as non-smoker, ex-smoker (previously smoking at any time in their lifetime) and current smoker. Data on alcohol consumption were collected during the previous 12 months and the subjects were classified as non-consumer (no alcohol consumption in the last 12 months), ex-consumer (alcohol had been consumed during the last 12 months but the patient had stopped consuming by the time of the interview) and current consumer. Drug use was defined as use of any drug in the lifetime. Other symptoms were all reported symptoms apart from the classic symptoms (a cough with or without blood, fever, weight loss, fatigue, night sweats, and shortness of breath). The body mass index (kg/m^2^) on the date of the interview was classified as underweight <18.50, normal range 18.50-24.99, and overweight ≥25.00.

The study was approved by the Ethics Committee of Teaching Public Health Institute of Split and Dalmatia County and all patients provided informed written consent.

### Inclusion and exclusion criteria

Cases that were detected through passive case-finding were defined as all adults (aged 15 years and older) with culture-confirmed pulmonary tuberculosis. TB patients who were identified during contact tracing, patients who had no symptoms before the diagnosis (e.g. identified during preoperative procedures), and patients with a previous history of TB treatment were excluded. The said patients had already had TB data recorded in their medical records and it was reasonable to assume that their physicians would have had a higher degree of suspicion when TB symptoms presented again. Such doctors would have thus been more alert and would have acted accordingly.

### Definitions

‘Health system delay’ was defined as the number of days from the first consultation with a physician (either primary care or other) to the initiation of anti-TB treatment. In the absence of a standard definition, ‘long health system delay’ was defined as a period exceeding the median delay observed in the study population, while ‘extreme delay’ was defined as a period exceeding the 75^th^ percentile delay. We did not separate diagnostic delay from treatment delay since it is standard practice in Croatia to start anti-TB therapy immediately following diagnosis.

### Data analysis

It was estimated that 160 participants (80 per group) should be included in this study to achieve an 80% power to detect an odds ratio of 2.5 with an alpha of 0.05. The median and 75^th^ percentile delays were calculated for all patients and all variables. We compared the distribution of delay ≤ median (or the 75^th^ percentile) with delay > median (or the 75^th^ percentile) by performing the Pearson Chi-squared test for all variables. The association of long and extreme delays with different variables was estimated using the crude odds ratios (OR) and 95% confidence intervals (CI). To identify factors independently associated with long and extreme health system delays, a multivariate logistic regression analysis was performed. All variables significant at a univariate level, as well as age groups, were included in the initial logistic regression model. The backward elimination procedure was used to identify the variables remaining in the final model and in the model-building strategies proposed by Hosmer and Lemeshow [15]. All analyses were conducted using Statistica 8.0 computer software (StatSoft Inc., Tulsa, USA). The level of statistical significance was defined as p < 0.05.

## Results

A total of 300 subjects were potentially eligible for this study. Among them, 41 patients had no clearly defined symptom onset or no symptoms before the diagnosis, 5 were identified during contact tracing, and 11 patients had a previous history of TB treatment. A total of 243 subjects were found to be eligible, two of whom refused to participate. A total of 241 participants were interviewed, 134 (56%) males and 107 (44%) females. The median health system delay was 15 days while the 75^th^ percentile was 42 days (the 5^th^ and 95^th^ percentile being 1 and 105 days respectively).

Within 10 days of their first visit to a health care facility, 99 (40.9%) cases were diagnosed, 29 (12%) within 11-20 days, 42 (17.4%) in the fourth week, while 71 (29.3%) remained undiagnosed for more than 30 days after the initial health care visit.

Descriptive information for the statistically significant and other epidemiologically relevant variables, such as demographic, socioeconomic and high-risk life style factors (Table 1), as well as diagnostic factors (Table 2), were presented. The symptoms were reported in various combinations, with a cough (73%) being the most frequently noted, followed by fatigue (65%) and weight loss (57%).

**Table 1:** Health system delay by demographic and socioeconomic factors and a high-risk life style

**Variable**	**Number**	**Median(days)**	**No (%) of participants with long delay**^**a**^	**No (%) of participants with extreme delay**^**b**^
Gender	Total 241			
Male	134	15	53 (39.6)	26 (19.4)
Female	107	28	62 (57.6) ^c^	33 (30.8) ^d^
Age groups	Total 241			
15-34	49	18	25 (51)	10 (20.4)
35-64	131	15	58 (44.3)	32 (24.4)
65+	61	21	32 (52.5)	17 (27.9)
Place of birth	Total 241			
Croatia	184	15	87 (47.3)	42 (22.8)
Foreign born	57	15	28 (49.1)	17 (29.8)
Level of education	Total 241			
No schooling or only elementary school	116	21	61 (52.6)	33 (28.4)
Secondary school	102	15	44 (43.1)	20 (19.6)
Higher education	23	15	10 (43.5)	6 (26.1)
Household contact	Total 241			
Yes	47	8	18 (38.3)	8 (17.0)
No	194	16.5	97 (50.0)	51 (26.3)
Contact outside the household	Total 241			
Yes	84	21	43 (51.2)	22 (26.2)
No	157	15	72 (45.9)	37 (23.6)
Smoker	Total 240			
Non-smoker	76	21	43 (56.6)	22 (28.9)
Ex-smoker	80	15	36 (45.0)	20 (25.0)
Current smoker	84	15	36 (42.9)	17 (20.2)
Alcohol consumption	Total 241			
Non-consumer	104	21	55 (52.9)	30 (28.8)
Ex-consumer	28	15	12 (42.9)	6 (21.4)
Current consumer	109	15	48 (44.0)	23 (21.1)
Ever used drugs	Total 241			
Yes	13	30	9 (69.2)	5 (38.5)
No	228	15	106 (46.5)	54 (23.7)

^a^ exceeding the median overall delay (15 days).

^b^ exceeding the75^th^ percentile overall delay (42 days).

^c^ significance levels of p < 0.05 for univariate logistic regression using the median (15 days) as a cut-off.

^d^ significance levels of p < 0.05 for univariate logistic regression using 75^th^ percentile (42 days) as a cut-off.

**Table 2:** Health system delay by diagnostic factor

**Variable**	**Number**	**Median (days)**	**No (%) of participants with long delay**^**a**^	**No (%) of participants with extreme delay**^**b**^
Previous hospitalisation	Total 241			
Yes	146	15	69 (47.3)	41 (28.1)
No	95	15	46 (48.4)	18 (18.9)
Comorbidity	Total 241			
Yes	101	18	51 (50.5)	28 (27.7)
No	140	15	64 (45.7)	31 (22.1)
Mantoux test results	Total 241			
Positive	222	15	107 (48.2)	4 (21.1)
Negative	19	15	8 (42.1)	55 (24.8)
Cavity on chest radiography	Total 241			
Yes	64	15	29 (45.3)	13 (20.3)
No	177	15	86 (48.6)	46 (26.0)
Specimen	Total 241			
Sputum	198	15	91 (46.0)	46 (23.2)
Other	43	24	24 (55.8)	13 (30.2)
Sputum smear-positive	Total 241			
Yes	166	15	68 (41.0)	29 (17.5)
No	75	30	47 (62.7) ^c^	30 (40.0) ^d^
Cough	Total 241			
Yes	176	15	84 (47.7)	40 (22.7)
No	65	15	31 (47.7)	19 (29.2)
Cough with blood	Total 241			
Yes	42	7	18 (42.9)	9 (21.4)
No	199	15	97 (48.7)	50 (25.1)
Fever	Total 241			
Yes	115	15	49 (42.6)	24 (20.9)
No	126	21	66 (52.4)	35 (27.8)
Weight loss	Total 241			
Yes	138	15	65 (47.1)	28 (20.3)
No	103	15	50 (48.5)	31(30.1)
Fatigue	Total 241			
Yes	156	15	71 (45.5)	34 (21.8)
No	85	21	44 (51.8)	25 (29.4)
Night sweats	Total 241			
Yes	112	15	46 (41.1)	21 (18.8)
No	129	28	69 (53.5)	38 (29.5)
Shortness of breath	Total 241			
Yes	73	15	31 (42.5)	18 (24.7)
No	168	16.5	84 (50.0)	41 (24.4)
Other symptoms	Total 241			
Yes	28	30	19 (67.9) ^c^	7 (25.0)
No	213	15	96 (45.1)	52 (24.4)
Body mass index ^e^	Total 237			
Underweight	31	15	15 (48.4)	6 (19.4)
Normal	156	15	75 (48.1)	44 (28.2)
Overweight	50	15	24 (48.0)	9 (18.0)

^a^ exceeding the median overall delay (15 days).

^b^ exceeding the 75^th^ percentile overall delay (42 days).

^c^ significance levels of p < 0.05 for univariate logistic regression using the median (15 days) as a cut-off.

^d^ significance levels of p < 0.05 for univariate logistic regression using the 75^th^ percentile (42 days) as a cut-off.

^e^ on the date of the interview.

When using the median as a cut-off to define long delay, female patients (p = 0.005, OR = 2.11, 95% CI 1.256-3.531), negative sputum smears (p = 0.002, OR = 2.42, 95% 1.381-4.239) and any other symptoms apart from those presented in the results (cough, cough with blood, fever, weight loss, fatigue, night sweats and shortness of breath) (p = 0.027, OR = 2.57, 95% CI 1.113-5.947) were found to be statistically significant. Other symptoms were independently associated with delay, with or without classic symptoms. Variables associated with long delay in the multivariate analysis were the female gender (p = 0.008, aOR = 2.06, 95% CI 1.213-3.517) and a negative sputum smear (p = 0.003, aOR = 2.38, 95% CI 1.341-4.236) with other symptoms (p = 0.037, aOR = 2.50, 95% CI 1.055-5.940) (Table 3).

**Table 3:** **Logistic regression **^**a **^**analysis of a long health system delay **^**b**^

	**Univariate analysis**		**Multivariate analysis**	
** Variable**	**p value**	**OR (95% CI)**	**p value**	**OR (95% CI)**
Gender				
Male		1		1
Female	0.005	2.11 (1.256-3.531)	0.008	2.06 (1.213-3.517)
Age groups				
15-34		1		1
35-64	0.420	0.76 (0.395-1.472)	0.96	0.98 (0.483-1.991)
65+	0.881	1.06 (0.499-2.248)	0.811	1.10 (0.499-2.432)
Sputum smear				
Positive		1		1
Negative	0.002	2.42 (1.381-4.239)	0.003	2.38 (1.341-4.236)
Other symptoms				
No		1		1
Yes	0.027	2.57 (1.113-5.947)	0.037	2.50 (1.055-5.940)

^a^ variables significant in the univariate logistic regression and age groups were included in the multivariate logistic regression. Statistical significance was taken at p < 0.05.

^b^ a long delay was considered to be above the median delay (15 days).

OR = Odds Ratio, CI = Confidence Interval.

When using the 75^th^ percentile to define extreme delay, the female gender (p = 0.042, OR = 1.85, 95% CI 1.024-3.352) and a negative sputum smear (p < 0.001, OR = 3.15, 95% CI 1.709-5.805) were both significant, while only a negative sputum smear (p < 0.001, OR = 3.11, 95% CI 1.680-5.770) remained significant in the multivariate analysis (Table 4).

**Table 4:** **Logistic regression **^**a **^**analysis of an extreme health system delay **^**b**^

	**Univariate analysis**		**Multivariate analysis**	
** Variable**	**p value**	**OR (95% CI)**	**p value**	**OR (95% CI)**
Gender				
Male		1		1
Female	0.042	1.85 (1.024-3.352)	0.056	1.81 (0.986-3.344)
Age groups				
15-34		1		1
35-64	0.571	1.26 (0.566-2.808)	0.261	1.63 (0.695-3.825)
65+	0.368	1.51 (0.617-3.677)	0.461	1.42 (0.561-3.579)
Sputum smear				
Positive		1		1
Negative	<0.001	3.15 (1.709-5.805)	<0.001	3.11 (1.680-5.770)

^a^ variables significant in the univariate logistic regression and age groups were included in the multivariate logistic regression. Statistical significance was taken at p < 0.05.

^b^ an extreme delay was considered to be above the 75^th^ percentile delay (42 days).

OR = Odds Ratio, CI = Confidence Interval.

## Discussion

The present study is one of the few studies of tuberculosis delay performed in countries with an intermediate burden of TB and the first one exploring health system delays in Croatia. We found that the median health system delay was 15 days. Numerous studies conducted elsewhere report longer delays [2,12,16-22], although a few studies show shorter delays [13,23-25].

Among demographic factors, only the female gender was found to be significantly associated with both a long and extreme delay. This finding was also reported in other countries [18,22,26-28], usually without proper explanation. In some of these countries (Uganda, Bangladesh) [22,26], cultural factors which might not have been relevant for Croatian females were likely to be involved. In our study, TB was clearly suspected and investigated more readily among men, although women did not seek care any later than men, as had previously been thought [8]. Therefore, this interesting finding warrants further investigation. Although it cannot be explained why women experienced delay in being treated, it is clear that women with symptoms suggestive of TB should receive more attention after seeking care.

The symptoms most frequently reported by patients were a cough, fatigue and weight loss. Unexpectedly, median delays were the same or similar both in the case of having one of the symptoms or having none. In addition, the presence of other symptoms was significantly associated with a long delay. Symptoms, either usual or not, are obviously not thought to be alarming and/or TB-related.

One of the challenges of diagnosing TB in primary care is to distinguish the symptoms of TB from those of other common respiratory diseases especially common in settings where community-acquired pneumonia and other lung diseases are more frequent than TB [29]. However, physicians have to be aware that a certain number of patients seeking medical assistance with symptoms of respiratory tract infections may have TB. It has been estimated that, on average, each general practitioner/family practice physician in Croatia has one TB patient every two years [30]. Under these circumstances, it is easy to neglect many important issues in TB management and control, resulting from a lack of experience and expertise which had previously been found to be associated with health system delays [31]. There was also a significant association between the sputum-smear status and health system delay among patients with negative smears who had double the median, as recently reported [27,32,33]. Smear-negative TB was obviously more difficult to diagnose and may have required an assessment of the response to antibiotic treatment, as well as chest radiography. These findings also indicate the urgent need for a more robust diagnostic test for diagnosing smear-negative cases in a timely manner.

The findings of this study need to be interpreted in the light of certain limitations. There may be a recall bias with reference to the symptoms and the timeliness of the health providers consulted. However, the participants were included prospectively and interviewed within one month after the TB diagnosis was made. Moreover, interviewers had received special training in two-day courses for proper data collection and control through as many sources as possible. This approach is believed to have minimised the bias. Another limitation of this study was the lack of data on the first point of contact with health professionals consulted for TB symptoms. However, the Croatian health care system is geared towards general practitioners/family practice physicians providing health care irrespective of socioeconomic class. If necessary, patients are referred to a specialist and TB drugs are available on prescription, and only on prescription. The few exceptions to this rule (emergency care, private doctor visit) are not likely to have significantly influenced our results.

The strength of this study lies in the fact that it was purpose designed. Secondly, this study covered almost 50% of all Croatian TB cases during the preceding year and more than 50% of the overall Croatian population in randomly selected counties. The dual TB notification system (physician and laboratory notification) had been successfully implemented since 1998 and so it can confidently be assumed that all confirmed cases were included in this study. Moreover, a larger sample size or more participating counties might have caused an under- or overestimation of delay.

## Conclusions

Despite free health care being universally available in Croatia and notwithstanding the ready availability of health services, it is of great concern that almost 20% of the patients under study remained undiagnosed in the fourth week after the initial health care visit, while one month later, a high proportion of the participants (29.3%) were still a source of infection within the community. Our findings appear to indicate some groups of TB patients having experienced a health system delay mainly due to a lack of suspicion. Although the health system delay in this study was found to be shorter than in many other countries, we believe that it could have been even shorter or avoided altogether had appropriate TB investigation been conducted earlier.

### Policy implications

From a public health point of view, it is imperative that physicians be better trained in TB, especially in settings where TB incidence is decreasing continuously. Emphasis should be placed on general practitioners/family medicine physicians, since they are the pillar of the health system and since most patients first go to them for treatment. These measures may be useful in reducing the number of missed opportunities for diagnosis and should form part of an approach to eliminate TB in countries with an intermediate tuberculosis burden.

## Competing interests

The authors declare that they have no competing interests.

## Authors’ contributions

AJS produced the concept and design, participated in the collection, analysis and interpretation of data, and drafted the manuscript. RM contributed to the concept and design, analysis and interpretation of data, and critically revised the manuscript for important intellectual content. BB, KK, LJBI, JV, GPC, SMD participated in the data collection, and revised critically the manuscript for important intellectual content. IG and AS participated in the analysis and interpretation of data and critically revised the manuscript for important intellectual content. All authors read and approved the final manuscript.

## Authors’ information

All authors have been deeply involved in TB control in Croatia from different standpoints. AJS, BB, KK, LJBI, JV, GPC and SMD are regional TB managers, AS is a national TB manager. IG is an experienced pulmonologist and RM is an experienced epidemiologist with a strong scientific background. AJS is continuously conducting and publishing studies on different aspects of TB control.

## Pre-publication history

The pre-publication history for this paper can be accessed here:

http://www.biomedcentral.com/1471-2458/13/250/prepub
